# Is the High Healing Index a Complication of Progressive Long Bone Lengthening? Observations from a Cohort of 178 Children Treated with Circular External Fixation for Lower Limb Length Discrepancy

**DOI:** 10.3390/children10101586

**Published:** 2023-09-22

**Authors:** Alessandro Depaoli, Marina Magnani, Agnese Casamenti, Tosca Cerasoli, Marco Ramella, Grazia Chiara Menozzi, Marina Mordenti, Gino Rocca, Giovanni Trisolino

**Affiliations:** 1 Unit of Pediatric Orthopedics and Traumatology, IRCCS Istituto Ortopedico Rizzoli, 40136 Bologna, Italy; alessandro.depaoli@ior.it (A.D.);; 2 Department of Industrial Engineering, Alma Mater Studiorum University of Bologna, 40136 Bologna, Italy; 3 Unit of Rare Skeletal Disorders, IRCCS Istituto Ortopedico Rizzoli, 40136 Bologna, Italy

**Keywords:** Ilizarov, external fixator, limb lengthening, child, leg length discrepancy, healing index, complications

## Abstract

The use of external fixators (EFs) for lower limb lengthening is common for treating lower limb length discrepancy (LLD) in children. The concern at present revolves around extended treatment times, with some suggesting a healing index (HI) > 45 days/cm as a major complication. The aim of this study is to assess the factors affecting bone healing and treatment duration in children who undergo limb lengthening for LLD using circular EFs. A total of 240 lengthening procedures on 178 children affected by congenital or acquired LLDs (mean age at surgery 13.8 ± 2.8 years) were retrospectively evaluated. Complications according to Lascombes’ classification and treatment duration factors were analyzed. Mean HI was 57 ± 25 days/cm for the femur and 55 ± 24 days/cm for the tibia, with an HI > 45 days/cm in 64% of the procedures. A total of 189 procedures (79%) reported complications; 85 had an HI > 45 days/cm as the sole complication. While reducing the frame time is crucial, revising the classifications is necessary to avoid the overestimation of complications.

## 1. Introduction

Lower limb length discrepancy (LLD) and the associated deformities are common in pediatric orthopedics, representing a heterogeneous group of congenital and acquired pathologies that alter bone morphology [1]. Lower limb inequality up to 1.5 cm has a prevalence of 21–32% in the healthy adult population; thus, this condition is considered clinically relevant if LLDs are 2 cm or longer at the end of growth [2,3,4]. Clinical and radiographic assessments are essential to evaluate the entity of LLDs in children and to estimate their magnitude at skeletal maturity, by not only considering the final LLD predicted with one of several multiplier methods (such as the White–Menelaus formula, Paley multiplier, or Sanders multiplier), but also the impact of the specific etiology over the acceleration and deceleration of limb growth [5,6,7,8,9]. Children with predicted LLDs longer than 2 cm are at a higher risk of developing severe postural disorders, gait abnormalities, spinal deformities, pain, discomfort, functional limitations, reduced physical activity, and psychosocial problems [10].

The management of LLD often involves a multidisciplinary approach, including orthopedic specialists, physical therapists, and psychologists. Various treatments have been proposed depending on the magnitude of the LLD predicted at the end of growth. For minor leg discrepancies (less than 2 cm), non-operative treatment is typically recommended. For discrepancies between 2 and 4 cm, growth modulation (epiphysiodesis) with the shortening of the longer side should be considered. When the predicted discrepancy exceeds 4 cm, there is a consensus for lengthening the shorter side, potentially combined with contralateral epiphysiodesis [10,11]. Despite the increasing popularity of intramedullary lengthening nails, procedures in the pediatric population mainly rely on external fixators to avoid physeal damage, with the circular external fixator (Ilizarov or hexapodal) still being one of the most used items [12]. A circular external fixator (EF) serves as a limb distractor and stabilizer, allowing for progressive bone distraction, the multiplanar correction of bone deformities, and preserving peri-osseous tissues through the distraction osteogenesis [1].

Treatment with EFs is highly effective in achieving limb lengthening and deformity correction without the need to prohibit weight-bearing on the limb during the entire period in frame. However, it exposes patients to a high risk of complications, including infections, nerve palsies, muscle or joint stiffness, progressive joint instability, and fractures of the regenerate bone [13]. In recent years, several scoring systems have been proposed for grading lengthening-related complications [14,15,16]. More recently, Lascombes et al. proposed a new and comprehensive classification system based on the concept of the “triple contract” [15]. According to this new method, the success of a lengthening procedure depends on three factors: achieving the intended length gain, adhering to the treatment duration, and maintaining limb function. In particular, the time in frame is a non-negligible element of the “contract”, since it has a significant impact on health, quality of life, and social interactions. Lascombes et al. identified an HI > 45 days/cm as a major complication (grade IIIa) [17]. The HI represents the ratio between the total time of treatment (TTT, defined as the number of days between EF implant and removal) and the total lengthening outcome achieved, expressed in days/cm [18]. TTT and HI may be influenced by multiple factors; although, the precise contributions of these factors remain uncertain [19].

This study aims to analyze a cohort of children and adolescents who underwent limb lengthening using circular EF for LLD, assessing the factors influencing bone healing and treatment duration.

## 2. Materials and Methods

This retrospective study included pediatric and adolescent patients who underwent one or more lower limb lengthening procedures using an EF at a single institution between January 2009 and December 2021 (IRB number 578/2020/O). Patients < 18 years old with LLDs, undergoing unilateral lengthening of the lower limb with circular EFs, were included. The exclusion criteria were adult patients (>18 years old), bilateral lengthening procedures (e.g., achondroplasia), conditions other than LLD (e.g., acute fractures), patients treated with an intramedullary lengthening nail, and patients with incomplete or missing radiographic data.

The patient data were extracted from medical records, including sex, affected side, family history, underlying pathologies, comorbidities, and LLD etiology. Previous surgeries outside our institution were recorded, along with LLD extent, age at the first visit, and preparatory surgical interventions for the lengthening procedure (e.g., hemi-epiphysiodesis, corrective osteotomies, etc.).

The surgical protocol had a minimal variation between patients. The corticotomy level was chosen according to the patient’s characteristics after the implantation of the EF and was always performed with chisels. The associated articular bridging of the knee was performed on patients with severe knee instabilities. Lengthening was started one or two days after the operation at a rate of 0.5 mm two times per day and continued until the planned lengthening was achieved. Full weight bearing was allowed as tolerated by the patient. Regular clinical and radiographic follow-ups were maintained during the lengthening period. The patient was planned for an EF removal when at least three complete cortices had formed in the regenerate bone. All patients were protected with a cast or brace for four weeks after the EF removal.

**Figure 1:**
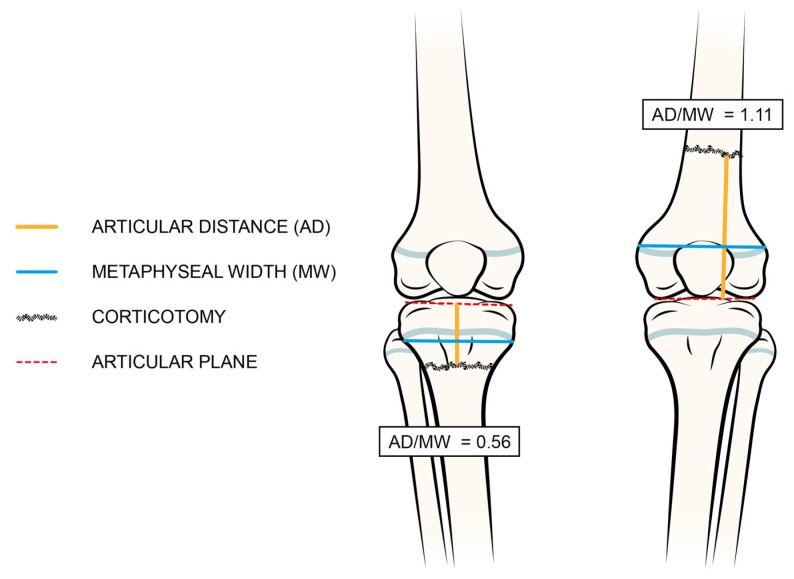
Technique of radiographic measurement of articular distance/metaphyseal width (AD/MW) ratio. The distance between the joint line (red dotted line) and the corticotomy level, depicted as a yellow line, is divided by the metaphyseal width, represented by a blue line.

Variables for each lengthening cycle and segment included LLD extent at the last clinical evaluation, age, height, weight, and body mass index (BMI) percentile for age and sex (with patients having a BMI ≥ 95 considered as obese), all at the time of the procedure; ongoing pharmacological therapies and the presence of deformities and/or joint instabilities adjacent to the segments being lengthened were assessed. Pre-operative ratios between the lengths of the hypometric segments and contralateral hypermetric segments were evaluated based on available radiographic images. Surgical details included the level of corticotomy, the need for joint bridging stabilization, and any additional procedures (hemi-epiphysiodesis, osteotomies, soft tissue procedures, etc.). In order to compare the corticotomy levels of patients with different ages and bone sizes, the distance between the corticotomy and joint plane (articular distance, AD) was divided by the maximum width of the respective metaphysis (metaphyseal width, MW) on post-operative anteroposterior radiographs (see details in Figure 1). This arbitrary measure, defined as the AD/MW ratio, is similar to the “rule of square”, widely described in traumatology to differentiate between metaphyseal or diaphyseal fractures [20]. Corticotomies were classified as diaphyseal if the AD/MW ratio exceeded 1, and as metaphyseal if the ratio fell between 0 and 1. The TTT and HI values were calculated for each procedure. All variables were recorded blindly by two independent authors (A.D. and A.C.).

Complications were assessed according to Lascombes’ classification [15], with an HI > 45 days/cm defined as a major complication (grade IIIa) but also considered separately from the other complications.

### Statistical Analysis

Statistical analyses were performed using STATA (version 17.0) based on the data collected in Excel 2021 (Microsoft Corporation, San Jose, CA, USA). The results were expressed as means (±standard deviation, SD) for continuous variables and as numbers with associated percentages for categorical variables. Normality was tested using chi-squared or Kolgomorov–Smirnov tests depending on the type of variable. Univariable and multivariable analyses with linear and logistic regressions were performed to assess the influence of baseline variables on bone healing (TTT and HI) and complications. Among the different groups and subgroups, a pairwise comparison of means was used to compare the continuous variables, using Bonferroni’s correction method for multiple comparisons, while contingency tables were compiled to evaluate the proportions’ relationships of categorical variables. A difference was considered statistically significant for a *p*-value less than 0.05.

## 3. Results

### 3.1. Patients Descriptives

**Figure 2:**
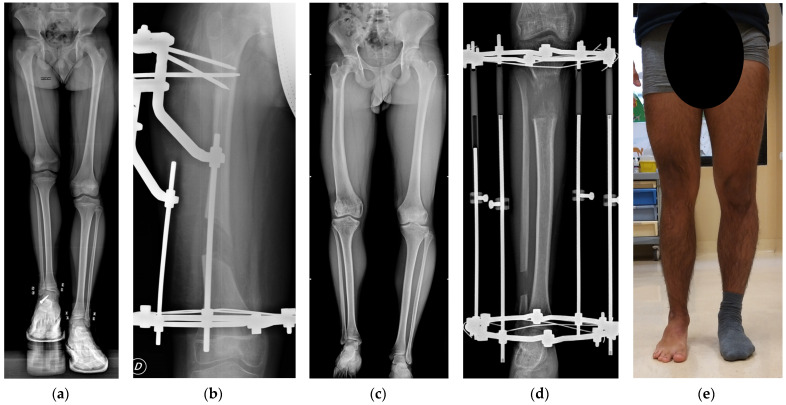
Case of LLD in congenital deficiency of the right femur in a male treated with two lengthening procedures with Ilizarov EFs. (**a**) Preoperative radiograph at 11 years and 10 months of age (LLD = −9 cm equal to 6% of height). The patient was treated for right flexible flat foot at the age of 9 years with subtalar joint arthroeresis with a non-absorbable talar screw that was removed before the first corticotomy procedure. (**b**) Radiograph after 50 days of femoral lengthening; after a few days, the patient achieved the planned lengthening of 5.5 cm and the EF was removed 220 days after the corticotomy (HI = 40 days/cm). (**c**) Pre-operative radiographs at 15 years and 10 months of age (LLD = −7.5 cm equal to 4.5% of height). (**d**) Radiograph after 60 days of lengthening, in which the patient achieved 6 cm of planned lengthening; patient developed contracture of Achilles tendon; EF was removed 229 days after the corticotomy (HI = 50 days/cm) and Achilles tendon contracture was treated with four-week cast immobilization and subsequently with physical therapy. (**e**) Clinical picture of lower limbs two years after tibial EF removal. At the last available follow-up, the patient did not complain about pain or limitation during sports, had a full range of motion in the right hip, knee, and ankle, with no signs of articular instability, and had a residual LLD of 1.5 cm, compensated with an insole.

A total of 178 patients (71 females and 107 males) were included in the study (see Table 1). The mean age at surgery was 13.8 ± 2.8 years. Among the patients, 26 (15%) were classified as obese. Simultaneous femur and tibia lengthening was performed on 37 patients (21%), and 25 patients underwent non-simultaneous lengthening, totaling 240 procedures (103 femurs and 137 tibiae, see Figure 2 for a case report of a patient treated with a lengthening procedure in each segment).

A total of 85 patients underwent prior surgery before lengthening, with 12 of them having procedures performed elsewhere (3 of which were lower limb lengthening procedures). At our institution, 78 patients received 126 procedures mainly for limiting the growth of the contralateral limb (epiphysiodesis) or for managing a malalignment or instability (hemi-epiphysiodesis, osteotomies, foot repositioning, and soft tissue procedures).

Most patients (84%) had congenital LLDs. Significant differences were observed in sex, age at surgery, and LLDs between congenital and acquired etiologies (Table S1 in the Supplementary Materials).

### 3.2. Radiographic Pre-Operative Variables

The mean pre-operative LLD was 6.5 ± 3.1 cm, predominantly affecting the tibia (43 patients) with 13.3% ± 7.8% shortening, femur (38 patients) with 11.2% ± 6.4% shortening, and mixed LLDs (97 patients) with 19.1% ± 10.9% of entire limb shortening (tibial shortening of 9.5% ± 7.7% combined with femoral shortening of 10.0% ± 7.0%).

### 3.3. Surgical Parameters and Outcomes

Most segments (230 cases) were lengthened using traditional Ilizarov circular EFs, while hexapod EFs were employed in 10 segments. Corticotomies were mainly diaphyseal in the femur (85%) and metaphyseal in the tibia (88%). The mean AD/MW ratios were 1.5 ± 0.5 (0.6–2.7) and 0.7 ± 0.2 (0.4–1.9) in the femur and tibia, respectively.

Mean TTT was 257 ± 116 days for the femur and 254 ± 82 days for the tibia. Mean HI was 57 ± 25 days/cm (range: 24–200) for the femur and 55 ± 24 days/cm (range: 25–157) for the tibia, without significant differences between the two bone segments (*p* = 0.58). The details of HI values by etiology are reported in Table 1. A total of 64% of the lengthening procedures had an HI > 45 days/cm. The simultaneous lengthening of both the femur and tibia resulted in an increased HI of 8 ± 4 days/cm (95% C.I. 1–15 days/cm) compared to single segment lengthening (*p* = 0.029). The HI was not statistically associated with LLD etiology (details in Table S2 in the Supplementary Materials).

To evaluate the impact of the AD/MW ratio on HI, we performed a visual analysis of their distribution in a two-way graph (see Figure S2 in the Supplementary Materials). The HI in tibial lengthening was slightly influenced by age at surgery (Spearman’s rho = 0.38; *p* < 0.005) and the AD/MW ratio (Spearman’s rho = −0.30; *p* < 0.005) (see details in Table S4 and Figure S3 in the Supplementary Materials). In the multivariate regression analysis, only age at surgery had a statistically significant impact on the HI in tibial procedures (R^2^ = 0.08, *p* = 0.006). Femoral HI was not affected by age at surgery and the AD/MW ratio.

There were no statistically significant differences in the HI between the first and second lengthening procedures in the same patient, nor in the HI based on the lengthened segment (femur or tibia).

### 3.4. Complications

A total of 51 procedures (21%) had no complications, while 85 (35%) reported an HI > 45 days/cm as the only complication. The remaining 104 lengthening procedures (43% of the entire case series) exhibited a total of 198 complications. Among these 104 procedures showing at least one complication other than a high HI, 30 presented only one complication, 54 had two complications, and 20 procedures had three complications (details in Table 2). 

The number of complications per procedure weakly correlated with the age at surgery (Spearman’s rho = 0.12; *p* = 0.073). The rate and type of complications were not influenced by the AD/MW ratio, sex, side, segment, congenital or acquired etiologies, or single or multisegmental lengthening (see details in Table S3 in the Supplementary Materials). Despite three procedures with HI values between 45 and 59, tibial lengthening in posteromedial bowing showed no other complications (0% of complications other than an HI > 45 days/cm, 97.5% one-sided C.I. = 0–37%), significantly less than all other etiologies of LLD (*p* = 0.034). Lengthening procedures in genetic skeletal disorders showed the highest rate of complications (13/22 procedures with a complication rate of 59%).

## 4. Discussion

The primary aim of this study was to investigate the factors influencing bone healing and treatment duration in lengthening procedures for either congenital or acquired LLDs in children and adolescents.

A significant and unexpected finding emerged, demonstrating the average HI of 57 days/cm for the femur and 55 days/cm for the tibia. These values largely exceeded the commonly applied 45 days/cm threshold established by Lascombes et al. [15]. However, our findings are consistent with several studies that also report an HI > 45 days/cm in large cohorts of pediatric and adolescent patients [17,21,22,23,24]. In our case series, most of the procedures (64%, 155/240) did not respect the threshold of 45 days/cm of HI. This is a concerning result since a prolonged time of EF wear is currently considered a major complication. However, only five procedures with an HI > 45 days/cm were clinically defined as delayed consolidation from the medical charts and radiographs and required additional treatments.

The cut-off period of 45 days/cm was first proposed by Donnan et al., who arbitrarily considered an HI ≤ 45 days/cm as acceptable and an HI > 45 days/cm as poor. However, in their series of 57 lengthening procedures, the same authors reported a mean HI of 55.5 days/cm, consistent with our findings. Lascombes et al. confirmed this threshold as a severe complication, without adding additional proof of concept or evidence [15].

In our study, age at surgery, etiology, bone segment involved, and level of corticotomy had no or marginal effects on the HI. In particular, age at surgery showed a statistically significant impact on the HI in tibia lengthening, as already proven in other studies, but was irrelevant from a clinical point of view [19,25,26]. Nonetheless, our findings confirm that the HI is paradoxically higher when shorter lengthening is required (see Figure S1 in the Supplementary Materials). This well-known phenomenon has been already described and it is probably explained by the lengthening process itself that is composed by a lengthening period, which has a linear correlation with the lengthening required, and a maturation period (inherent with biological properties of the bone) much less influenced by the practice of lengthening [19,25]. Moreover, the HI is calculated considering the TTT, which corresponds to the duration between the implantation and removal of the EF. Based on our observations, this timeframe might have been inadvertently extended by up to 30 days due to the scheduling of EF removal and the time spent on the waiting list.

For these reasons, many authors more properly define the external fixator index (EFI) rather than the HI [24]. Although the HI allows us to compare the different techniques, such as intramedullary lengthening nails versus EFs, for patients treated with EFs, the time in frame is the major concern. Therefore, several authors proposed alternative strategies for reducing the EFI [24]. The association of EF with an internal fixation system (elastic stable intramedullary nails, rigid intramedullary nails, and MIPO plates) has been widely reported, with a significant reduction in the EF bearing time in all cohorts. Other authors demonstrated that physical and/or biological bone stimulations with low-intensity pulsed ultrasounds (LIPUSs), pulsed electromagnetic fields (PEMFs), bone marrow concentrate (BMC), or platelet-rich plasma (PRP) injections in the regenerate area after distraction osteogenesis may all significantly decrease the time spent in EFs [27,28]. All these strategies can be truly effective; however, the potentially increased risks of additional surgical procedures and the extra costs must be taken into consideration. More easily, an optimized treatment protocol that reduces the time spent between scheduling and performing the EF removal by as much as possible can be beneficial as well in reducing the EFI.

The high number of complications reported in the study mostly occurred due to the inclusion of an HI > 45 days/cm in the definition of complications according to Lascombes’ classification [15]. This finding confirms the safety of the procedure but also highlights the need to reduce the HI. Apart from the increased HI, we reported 133 complications overall in 104/240 lengthening procedures (43%). Among them, 55 complications were considered grades III or IV according to Lascombes et al. We reported 31 cases of joint stiffness, a complication possibly influenced by the duration of EF wear. However, our data did not statistically demonstrate this assumption. On the other hand, we reported only four cases of fractures of the regenerate bone after EF removal (1.7%). Three cases were treated with cast immobilizations, while only one case experienced a significant loss of lengthening requiring further surgery. The high values of HI and TTT can partially justify the very low rate of fractures of regenerate bones. The etiology did not show a significant influence on the complication rate and severity, probably due to the low number of cases in each subgroup. The very low prevalence of complications in eight patients treated for a congenital posteromedial bowing of the tibia was encouraging; however, many larger case series reported relevant incidences of complications in this subgroup [29,30].

Our study presented some limitations, such as the retrospective design and the numberless etiology included, with some conditions reporting less than three cases. This can affect the statistical inference between the etiology and outcomes. Moreover, our study did not include data about the clinical and functional results. Further research is still ongoing in our department for developing prospective and retrospective surgical registries for limb lengthening, which include disease-specific patient-reported outcomes.

## 5. Conclusions

This study confirms that a circular EF is an effective procedure for treating LLDs in children and adolescents. The burden of complications is relevant; however, our results suggest that an HI < 45 days/cm can be misleading if considered as a major complication. Additional research and further evidence must be produced, especially for identifying the most appropriate method and threshold for establishing the excessive duration of treatment as a potential negative result.

## Figures and Tables

**Table 1:** Patients’ descriptives and surgical outcomes by etiology. N = number; M = male; F = female; HI = healing index; TTT = total time of treatment; CFD = congenital femoral deficiency; DDH = developmental dysplasia of the hip; CCF = congenital clubfoot; CPMBT = congenital posteromedial tibial bowing; NF1 = neurofibromatosis type 1; CPT = congenital pseudoarthrosis of tibia; MHE = multiple hereditary exostoses; LCPD = Legg-Calvè-Perthes disease.

	N Patients (%)	N Procedures (%)	M:F	Mean Age at Surgery (Years)	Mean Preoperative LLD (cm)	Mean HI (Days/cm)	Mean TTT (Days)	% Procedures with One or More Complications
**Congenital causes**	**150 (84%)**	**204 (85%)**	**1.7:1**	**13.5 ± 2.9**	**6.6 ± 3.1**	**55 ± 24**	**257 ± 98**	**78%**
				**(5.2–17.5)**	**(2.0–20.0)**	**(24–200)**	**(106–983)**	
Idiopathic	29 (16%)	35 (15%)	1.8:1	14.5 ± 1.8	5.4 ± 2.6	62 ± 22	258 ± 80	89%
				(10.4–17.3)	(2.5–13.5)	(35–151)	(170–604)	
Proximal limb *CFD*	50 (28%) *46*	66 (28%) *62*	*1.9:1*	*13.7* ± *2.7* *(6.9–17.4)*	*6.9* ± *3.4* *(2.0–20.0)*	*59* ± *30* *(24–200)*	*267* ± *138* *(123–983)*	*77%*
*Hypoplasia in DDH*	*4*	*4*	*1:1*	*14.6* ± *2.1* *(11.7–16.3)*	*6.3* ± *2.5* *(4.0–9.0)*	*64* ± 6 *(59–71)*	*272* ± *72* *(208–351)*	*75%*
Distal limb *Fibular hemimelia*	53 (30%) *24*	78 (32%) *45*	*2:1*	*10.4* ± *3.4* *(5.2–15.7)*	*8.3* ± *3.0* *(3.0–16.0)*	*50* ± *22* *(25–157)*	*258* ± *93* *(106–705)*	*71%*
*Tibial hemimelia*	*15*	*18*	*3:1*	*14.3* ± *1.8* *(9.3–16.4)*	*6.6* ± *2.5* *(4.0–12.0)*	*55* ± *22* *(27–100)*	*249* ± *46* *(174–345)*	*83%*
*CPMBT*	*8*	*8*	*0.3:1*	*13.7* ± *2.2* *(8.4–15.1)*	*5.3* ± *1.4* *(4.0–8.5)*	*44* ± *8* *(35–59)*	*226* ± *43* *(157–287)*	*38%*
*Hypoplasia in CCF*	*6*	*7*	*2:1*	*15.3* ± *1.9* *(11.7–17.0)*	*4.3* ± *1.6* *(2.0–6.5)*	*56* ± *16* *(39–83)*	*237* ± *68* *(168–373)*	*71%*
Skeletal dysplasias	15 (8%)	22 (9%)	1.6:1	14.2 ± 3.1	7.4 ± 3.9	49 ± 16	249 ± 90	82%
*Ollier’s disease*	*7*			(7.7–17.4)	(3.0–15.0)	(29–87)	(159–491)	
*CPT without NF1*	*1*							
*CPT in NF1*	*2*							
*NF1 (without CPT)*	*1*							
*MHE*	*2*							
*Others*	*2*							
Hemi-hypertrophy	3 (2%)	3 (1%)	2:1	13.6 ± 3.6	4.1 ± 0.8	68 ± 49	250 ± 46	67%
				(9.6–16.6)	(3.5–5.0)	(31–123)	(206–298)	
**Acquired causes**	**28 (16%)**	**36 (15%)**	**0.8:1**	**15.2 ± 1.6**	**6.1 ± 2.7**	**60 ± 26**	**247 ± 96**	**86%**
				**(12.0–17.9)**	**(2.0–12.5)**	**(28–127)**	**(97–455)**	
Infections	10 (5%)	17 (7%)	1:1	14.6 ± 1.7	8.2 ± 2.7	67 ± 27	286 ± 120	94%
*Osteomyelitis*	*5*			(12.0–16.7)	(3.5–12.5)	(37–114)	(97–455)	
*Septic arthritis*	*5*							
Trauma (fractures, vascular deficits)	14 (8%)	15 (6%)	0.7:1	15.5 ± 1.5	5.4 ± 1.9	48 ± 13	212 ± 37	73%
				(12.0–17.9)	(3.0–10.0)	(28–64)	(172–299)	
Idiopathic conditions	2 (1%)	2 (1%)	0:2	14.8 ± 0.9	3.0 ± 1.4	87 ± 57	284 ± 139	100%
*Blount’s disease*	*1*			(14.2–15.5)	(2.0–4.0)	(46–127)	(185–382)	
*LCPD*	*1*							
Neoplastic conditions	2 (1%)	2 (1%)	1:1	16.1 ± 0.8 (15.5–16.6)	4 (3.5–4.5)	69 ± 23 (53–85)	169 ± 59 (127–210)	100%
**TOTAL**	**178** **(100%)**	**240** **(100%)**	**1.5:1**	**13.8 ± 2.8** **(5.2–17.9)**	**6.5 ± 3.1** **(2.0–20.0)**	**56 ± 24** **(24–200)**	**255 ± 98** **(97–983)**	**79%**

**Table 2:** Complications according to Lascombes’ classification. N = number; EF = externa fixator; GA = general anesthesia; HI = healing index. In this table, procedures with an HI > 45 days/cm are not considered. (*) = among 155 procedures with an HI > 45 days/cm, 85 only reported this complication. For the remaining 70 cases, only 5 procedures were clinically and radiographically diagnosed as delayed consolidation and required specific treatment.

Grade According to Lascombes’ Classification	N
**I**	**55**
Superficial infection requiring antibiotics	46
Swelling of the lower limb	3
Superficial thrombophlebitis	1
Temporary nerve palsy	5
**IIa**	**18**
Single pin breakage or detachment	10
Early union of regenerate	3
Revision of EF under GA	5
**IIb**	**0**
**IIIa**	**204**
HI > 45 days/cm	155 *
Joint stiffness	31
Fracture after EF removal	4
Non-union	6
Residual angular deformity requiring osteotomy	8
**IIIb**	**2**
Lengthening procedure interrupted and EF removed	2
**IVa**	**4**
Severe knee instability or subluxation	4
**IVb**	**0**
**Total**	**283**

## Data Availability

Data are available from the corresponding authors upon reasonable request.

## Supplementary Materials

The following supporting information can be downloaded at: https://www.mdpi.com/article/10.3390/children10101586/s1, Table S1: congenital versus acquired etiology comparison of baseline variables; Table S2: mean healing index (HI) by etiology groups and bone segments; Table S3: association between preoperative variables and rate of complications; Table S4 (a–c): Spearman’s rho correlation table between variables in overall procedures and in femur and tibia segments; Figure S1: distribution of healing index (HI) by lengthening achieved in centimeters; Figure S2: distribution of healing index (HI) by articular distance/metaphyseal width (AD/MW) ratio for tibial and femoral segments; Figure S3: distribution of healing index (HI) by age at surgery for tibial and femoral segments.
