# Supplementary material for: Is the High Healing Index a Complication of Progressive Long Bone Lengthening? Observations from a Cohort of 178 Children Treated with Circular External Fixation for Lower Limb Length Discrepancy
Source: Children (Basel). 2023 Sep 22;10(10):1586. doi: 10.3390/children10101586 (PMC10605005; doi:10.3390/children10101586)

SUPPLEMENTARY MATERIALS

Table S1. Congenital versus acquired etiology comparison of baseline variables. M = males; F = females.

	**Congenital**	**Acquired**	**p-value**
Sex	55 F, 95 M	16 F, 12 M	0.015
Age at first pediatric orthopedic evaluation (years)	8.0 ± 5.1	10.4 ± 3.6	0.0001
Age at surgery (years)	13.6 ± 2.9	15.2 ± 1.6	0.002
LLD pre-operative (cm)	6.6 ± 3.1	6.1 ± 2.7	0.0001

Table S2. Mean healing index (HI) by etiology groups and bone segments. HI = healing index; LLD = lower limb length discrepancy.

LLD etiology	Mean HI in Femur (days/cm)	Mean HI in Tibia (days/cm)
Idiopathic LLD	62 ± 17	61 ± 24
Congenital femur hypoplasia	59 ± 33	60 ± 22
Developmental hip dysplasia	64 ± 6	-
Fibular hemimelia	51 ± 11	49 ± 25
Tibial hemimelia	69 ± 19	54 ± 22
Congenital posteromedial bowing	-	44 ± 8
Pes equinovarus	-	56 ± 16
Skeletal dysplasias	55 ± 19	45 ± 13
Hemihypertrophy	-	68 ± 49
Bone infection	64 ± 27	72 ± 30
Post-traumatic LLD	43 ± 14	56 ± 6
Other acquired etiologies	66 ± 28	90 ± 52
**Total**	**57 ± 25**	**55 ± 24**

Table S3. Association between preoperative variables and rate of complications.

**Variables evaluated for influence on complications**	**p-value**
Sex	0.67
Side	0.51
Obesity	0.23
Segment	0.38
Congenital or acquired etiology	0.11
Multisegmental lengthening	0.76
Hip deformity	0.88
Knee deformity	0.16
Foot deformity	0.01
Fixator type (Ilizarov or hexapod)	0.82
Articular bridge fixation	0.58

Table S4 (a-c). Spearman's rho correlation table between variables in overall procedures (a) and in femur (b) and tibia (c) segments. TTT = total time of treatment; BMI = body mass index; AD/MW = articular-distance metaphyseal-width; LLD = lower limb length discrepancy.

OVERALL (a)

	HI	TTT	Age at surgery	Height	Weight	BMI percentile	AD/MW ratio	LLD pre-op ass	LLD%
TTT	**0.41*****								
Age at surgery	0.10	0.06							
Height	0.01	-0.08	**0.63*****						
Weight	0.08	-0.07	**0.44*****	**0.66*****					
BMI percentile	-0.01	**-0.19****	**-0.19****	-0.01	**0.60*****				
AD/MW ratio	-0.02	-0.08	-0.10	-0.02	-0.10	-0.04			
LLD pre-op ass	**-0.18****	**0.21****	**-0.37*****	**-0.23*****	**-0.24*****	-0.06	**0.16***		
LLD%	-0.12	**0.30*****	**-0.41*****	**-0.36*****	**-0.27*****	-0.06	**0.13***	**0.97*****	
Lengthening	**-0.42*****	**0.20***	**-0.32*****	**-0.25****	**-0.30*****	-0.11	**0.20***	**0.77*****	**0.69*****

FEMUR (b)

	HI	TTT	Age at surgery	Height	Weight	BMI percentile	AD/MW ratio	Femur%	-
TTT	**0.36*****								
Age at surgery	-0.03	-0.09							
Height	-0.16	**-0.28***	**0.62*****						
Weight	-0.03	**-0.28***	**0.36****	**0.66*****					
BMI percentile	0.02	**-0.25***	-0.18	0.05	**0.67*****				
AD/MW ratio	0.10	-0.05	-0.06	0.01	-0.02	-0.09			
Femur%	**0.20***	**-0.20***	**0.33****	**0.29****	**0.20***	**-0.19***	**0.27****		
Tibia%	-0.10	-0.03	**0.37*****	**0.32****	**0.18***	-0.08	-0.07	0.05	

TIBIA (c)

	HI	TTT	Age at surgery	Height	Weight	BMI percentile	AD/MW ratio	Femur%	-
TTT	**0.46*****								
Age at surgery	**0.20***	**0.18***							
Height	0.11	0.06	**0.63*****						
Weight	0.15	0.07	**0.48*****	**0.66*****					
BMI percentile	-0.01	**-0.16***	**-0.19***	-0.04	**0.56*****				
AD/MW ratio	**-0.17***	-0.05	**-0.18***	**-0.16***	**-0.17***	0.05			
Femur%	-0.08	0.01	**0.18***	0.09	0.05	-0.11	**-0.24****		
Tibia%	**0.32*****	**-0.18***	**0.30*****	**0.35*****	**0.35*****	**0.16***	-0.05	**-0.21***	

Figure S1. Distribution of healing index (HI) by lengthening achieved in centimeters. An overall polynomial trend line is depicted.

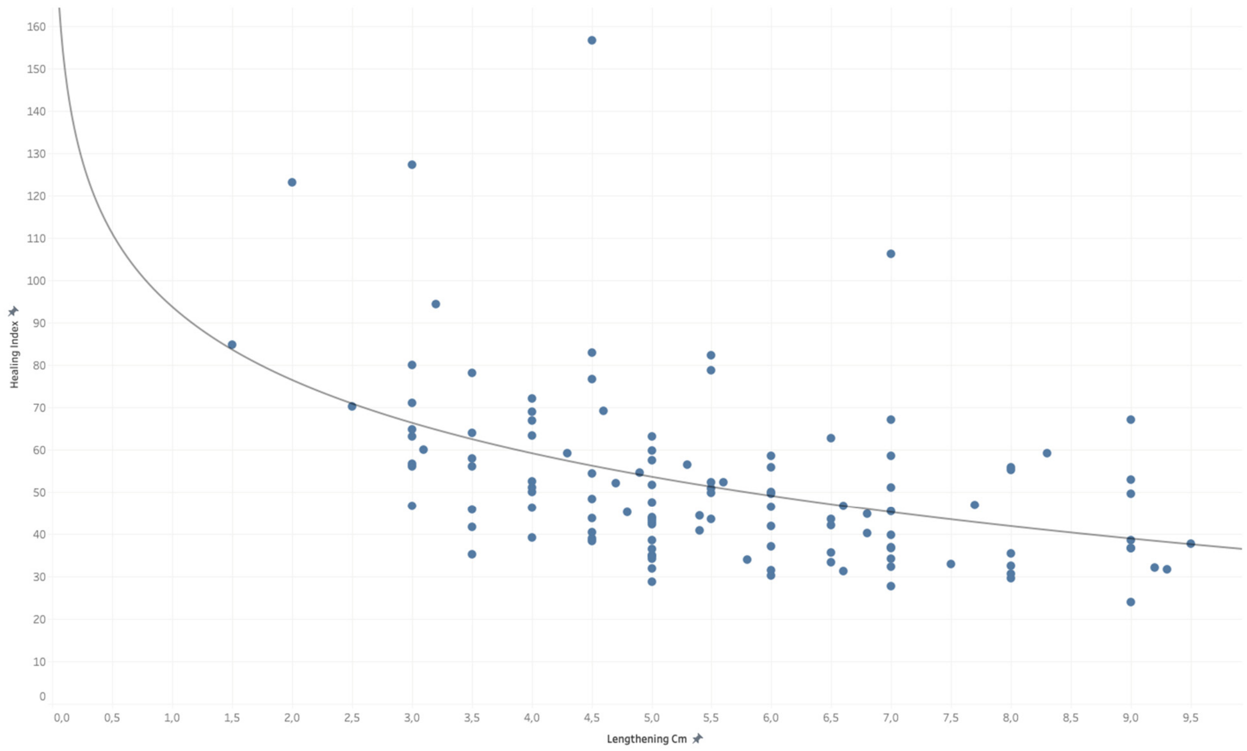

Figure S2. Distribution of healing index (HI) by articular-distance metaphyseal-width (AD/MW) ratio for tibial segments (green) and femoral segments (red). An overall polynomial trend line with confidence intervals is depicted.

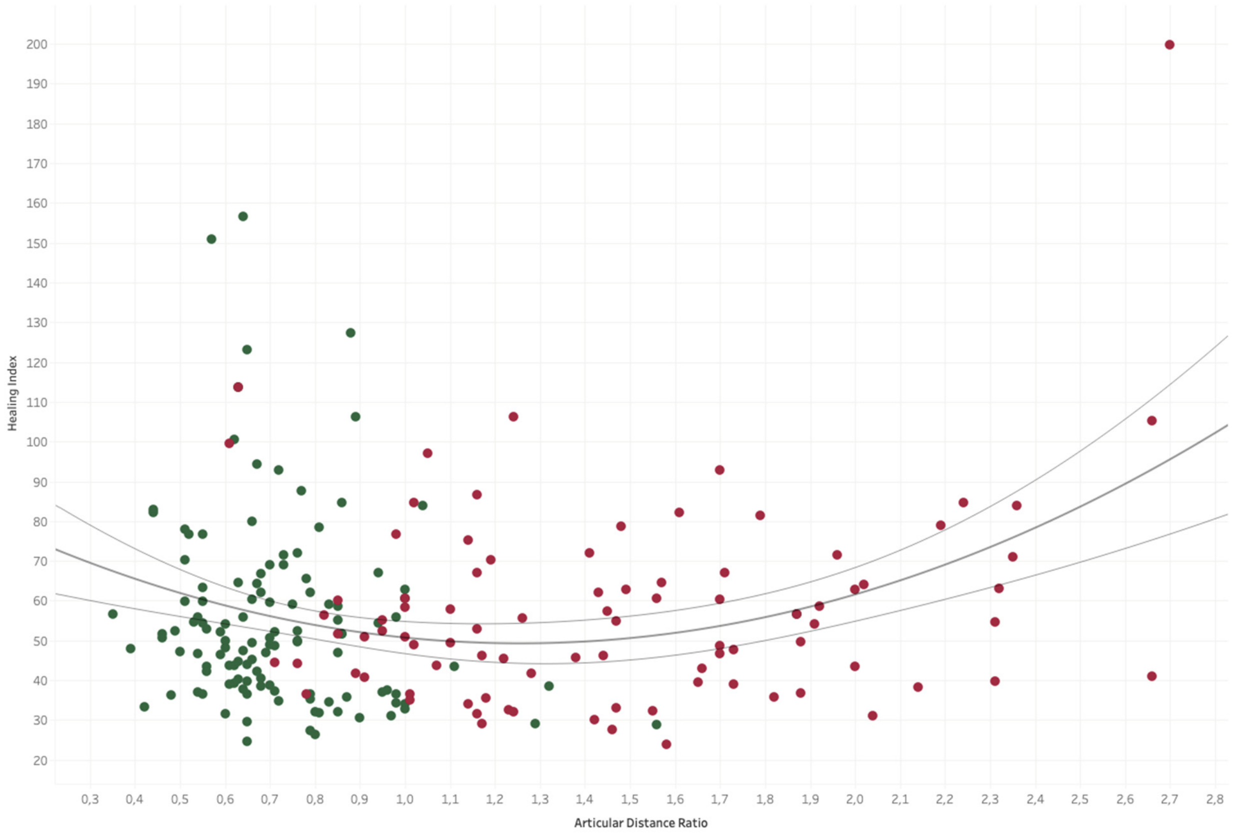

Figure S3. Distribution of healing index (HI) by age at surgery for tibial segments (green) and femoral segments (red). A slight reduction of HI was observed in younger patients only for tibial lengthening procedures (p = 0.036).

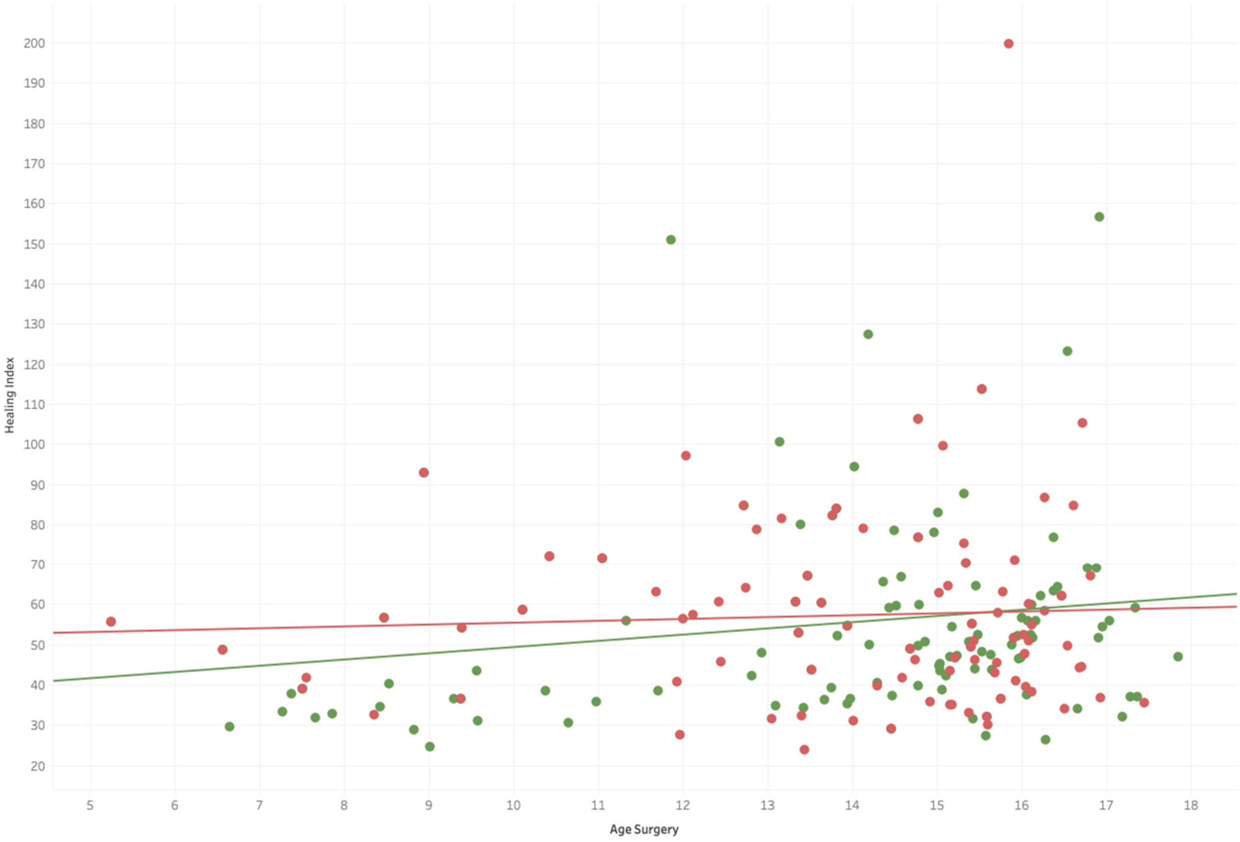

Supplement: Supplementary file 1 [file children-10-01586-s001.zip › children-2602087-supplementary.pdf]
